# Identification of rare coding variants associated with Kawasaki disease by whole exome sequencing

**DOI:** 10.5808/gi.21046

**Published:** 2021-12-31

**Authors:** Jae-Jung Kim, Young Mi Hong, Sin Weon Yun, Kyung-Yil Lee, Kyung Lim Yoon, Myung-Ki Han, Gi Beom Kim, Hong-Ryang Kil, Min Seob Song, Hyoung Doo Lee, Kee Soo Ha, Hyun Ok Jun, Byung-Ok Choi, Yeon-Mok Oh, Jeong Jin Yu, Gi Young Jang, Jong-Keuk Lee, The Korean Kawasaki Disease Genetics Consortium

**Affiliations:** 1 Asan Institute for Life Sciences, Asan Medical Center, University of Ulsan College of Medicine, Seoul 05505, Korea; 2 Department of Pediatrics, Ewha Womans University Hospital, Seoul 07985, Korea; 3 Department of Pediatrics, Chung-Ang University Hospital, Seoul 06973, Korea; 4 Department of Pediatrics, Daejeon St. Mary’s Hospital, College of Medicine, The Catholic University of Korea, Daejeon 34943, Korea; 5 Department of Pediatrics, Kyung Hee University Hospital at Gangdong, Seoul 05278, Korea; 6 Department of Pediatrics, Gangneung Asan Hospital, University of Ulsan College of Medicine, Gangneung 25440, Korea; 7 Department of Pediatrics, Seoul National University Children's Hospital, Seoul 03080, Korea; 8 Department of Pediatrics, Chungnam National University Hospital, Daejeon 35015, Korea; 9 Department of Pediatrics, Inje University Paik Hospital, Busan 47392, Korea; 10 Department of Pediatrics, Pusan National University Hospital, Busan 49241, Korea; 11 Department of Pediatrics, Korea University Guro Hospital, Seoul 08308, Korea; 12 Department of Pediatrics and Adolescent Medicine, Myongji Hospital, Goyang 10475, Korea; 13 Department of Neurology, Samsung Medical Center, Sungkyunkwan University School of Medicine, Seoul 04401, Korea; 14 Department of Pulmonary and Critical Care Medicine, Asan Medical Center, University of Ulsan College of Medicine, Seoul 05505, Korea; 15 Department of Pediatrics, Asan Medical Center, University of Ulsan College of Medicine, Seoul 05505, Korea; 16 Department of Pediatrics, Korea University Ansan Hospital, Ansan 15355, Korea

**Keywords:** association study, coronary artery aneurysms, Kawasaki disease, whole exome sequencing

## Abstract

Kawasaki disease (KD) is an acute pediatric vasculitis that affects genetically susceptible infants and children. To identify coding variants that influence susceptibility to KD, we conducted whole exome sequencing of 159 patients with KD and 902 controls, and performed a replication study in an independent 586 cases and 732 controls. We identified five rare coding variants in five genes (*FCRLA*, *PTGER4*, *IL17F*, *CARD11*, and *SIGLEC10*) associated with KD (odds ratio [OR], 1.18 to 4.41; p = 0.0027–0.031). We also performed association analysis in 26 KD patients with coronary artery aneurysms (CAAs; diameter > 5 mm) and 124 patients without CAAs (diameter < 3 mm), and identified another five rare coding variants in five genes (*FGFR4*, *IL31RA*, *FNDC1*, *MMP8*, and *FOXN1*), which may be associated with CAA (OR, 3.89 to 37.3; p = 0.0058–0.0261). These results provide insights into new candidate genes and genetic variants potentially involved in the development of KD and CAA.

## Introduction

Kawasaki disease (KD) is an acute, self-limited vasculitis that occurs predominantly in infants and young children [1]. KD is often complicated by coronary artery aneurysms (CAAs), which occur in approximately 15%–25% of untreated children and 3%–5% of children treated with high-dose intravenous immunoglobulin (IVIG) [2-4]. Although the pathogenesis of KD remains unknown, it has been postulated that KD is an abnormal immunological reaction to an infection in genetically susceptible individuals [5,6].

Many complex traits are shaped by intricate interplay between common and rare genetic variants. Recently, genome-wide association studies (GWAS) of common single nucleotide polymorphisms (SNPs) have identified several variants associated with susceptibility to KD [7-11]. Although identification of these common variants has contributed somewhat to understanding the biology of KD, many complexities of the disease remain to be unraveled [12]. Furthermore, most GWAS-derived SNPs do not directly influence the disease trait. Rather, they are index markers in linkage disequilibrium with causative variants for the disease; hence, it is necessary to identify rare coding variants that affect susceptibility to KD using other approaches, such as next-generation sequencing technology. Whole exome sequencing (WES) is a powerful technique for identification of rare protein-coding variants. Here, we used WES and association analysis to identify the protein-coding variants that influence susceptibility to KD and CAA.

## Methods

### Subjects

Children with KD were recruited from 12 tertiary academic hospitals in Korea, all of which participated in the Korean Kawasaki Disease Genetics Consortium. All patients were diagnosed by pediatricians in accordance with the diagnostic criteria of the American Heart Association [13,14]. All patients received a single high-dose of IVIG (2 g/kg). Two-dimensional echocardiography results were interpreted by pediatric cardiologists, and coronary arteries were categorized as normal or abnormal (showing coronary artery lesions (i.e., dilation or aneurysm)). A total of 200 children with KD were initially included in the WES analysis. Our WES data were deposited to the Biobank for Health Sciences, Chungwon, Korea (http://nih.go.kr/biobank; accession number 2016-ER7401-00). For association analysis of WES data from KD cases, a total of 902 control WES or whole genome sequencing (WGS) data samples were obtained from Dr. Yeon-Mok Oh (Asan Medical Center, Seoul, Korea; WES data from 147 patients with chronic obstructive pulmonary disease), Dr. Byung-Ok Choi (Samsung Medical Center, Seoul, Korea; WES data from 258 patients with Charcot–Marie–Tooth disease), and the Biobank for Health Sciences (Center for Genome Science, Chungwon, Korea; WES data from 100 healthy subjects and WGS data from 397 healthy adults). For replication study, a total of 586 patients with complete KD were selected from our KD patient pool. The healthy adult control samples (n = 732) were obtained from the Biobank for Health Sciences (Chungwon, Korea). This study was approved by the institutional review board (IRB) at Asan Medical Center (IRB No. 2014-0823), and informed consent was obtained from the parents of patients with KD.

### Targeted capture and exome sequencing

WES was conducted using genomic DNA samples obtained from 200 children with KD. For exome sequencing, 3 μg genomic DNA was initially sheared into 150-base-pair (bp) fragments for shotgun library construction. Fragmented DNA from each sample was captured using the SureSelect Human All Exon V5 Kit (Agilent Technologies, Santa Clara, CA, USA), designed to cover 50 Mb of human genomic sequences corresponding to the NCBI Consensus Coding Sequence database. Each captured library was sequenced at Macrogen (Seoul, Korea) using an Illumina HiSeq 2500 instrument (Illumina, San Diego, CA, USA), according to the manufacturer’s instructions.

### Read mapping and variant analysis

Sequence reads in FASTQ format were aligned to a human genome reference sequence (GRCh37) using Burrows–Wheeler Aligner (BWA) software [15]. Duplicate reads were marked in BAM files using Picard MarkDuplicates (https://broadinstitute.github.io/picard/). Local realignment, base quality recalibration, variant calling, joint genotyping, and variant quality score recalibration and filtration were applied using Genome Analysis ToolKit (GATK) v3.7 (http://software.broadinstitute.org/gatk/). Variant annotation was performed using SnpEff [16].

### Genotyping

Genotyping for the replication study was performed on the high-throughput Fluidigm EP1 system (Fluidigm Corp., South San Francisco, CA, USA), using the Fluidigm SNPType assay platform, and nanofluidic 96.96 and 48.48 Dynamic Array Integrated Fluidic Circuits (Fluidigm). Genotype calls were made using the Fluidigm SNP Genotyping Analysis program (Fluidigm). All but one of the selected SNPs were genotyped for replication. The overall genotyping success rate was 99.5%.

### SNP filtering

After WES, a total of 870,062 SNPs were identified from 200 KD cases and 902 controls. To filter the SNP markers, we excluded 337,282 SNP markers with missing call rates > 3%, 138,329 monomorphic markers, and 3,404 multiallelic markers in patients with KD. As coding variants that cause amino acid changes may be hypothesized to confer larger phenotypic effects, we also excluded 313,470 intergenic, intronic, untranslated-region, and synonymous SNPs. After filtering, 77,577 protein-altering variants remained, among which 75,365 were missense, 1,448 were nonsense, and 764 were in splice-acceptor/donor sites. Next, we excluded 3,209 SNPs with missing call rates > 5% (slightly looser threshold due to heterogeneous control data compared to case data) in control data. Finally, a total of 74,368 SNPs were included in the association analysis.

### Statistical analysis

All statistical analyses were performed using PLINK software v1.07 (http://pngu.mgh.harvard.edu/~purcell/plink/) [17]. The chi-square or Fisher’s exact tests (when the expected cell count was less than five) were used to compare SNP allele frequencies between cases and controls to test for associations with KD and CAA.

## Results

### Associations with KD

WES was initially performed using samples from 200 children with KD, of which 159 had complete KD with a fever lasting ≥ 5 days and > 4 of the principal symptoms of KD. Therefore, we performed association analysis of 159 complete KD cases and 902 controls. For the replication study, we selected 12 coding SNPs with significant associations with KD susceptibility (chi-square test p < 0.001) and immune-related functions. Extremely rare variants are more likely to be deleterious; thus we also selected an additional 51 immune-related rare coding SNPs detected in case samples only, or with odds ratios (ORs) ≥ 3 in case-control comparisons. We tested these 63 SNPs in 586 patients with complete KD and 732 healthy controls using the Fluidigm SNPType assay platform; however, none showed statistical significance (p > 0.05) due to their very low frequencies in both case and control samples; however, among them, five coding SNPs (rs2275603 in *FCRLA*, rs755244149 in *PTGER4*, rs117796773 in *IL17F*, rs41493047 in *CARD11*, and rs201376644 in *SIGLEC10*) showed nominal significances (OR = 1.18 to 4.41, p = 0.0027–0.031) in combined analysis of data generated by WES and Fluidigm SNPType assay (Table 1).

### Associations with CAA

Of the 200 children with KD, 74 had coronary artery lesions. To investigate genetic loci that affect the development of CAAs in KD, we initially stratified our KD samples by CAA size, according to the American Heart Association statement [13], as follows: small, 3 to <5 mm; medium, 5 to 8 mm, or giant, >8 mm. Next, we performed association analysis in 124 KD patients without CAAs (<3 mm) and 26 KD patients with medium or giant aneurysms that showed more extreme phenotypes (internal lumen diameter of coronary arteries > 5 mm). For the replication study, we selected five SNPs with significant associations with CAAs (chi-square test p < 0.001) and CAA-related functions, and tested them in 420 patients with KD without CAAs and 19 with CAAs (diameter > 5 mm). Although no SNP showed significant results in the replication study, five coding variants (rs148721785 in *IL31RA*, rs201812753 in *FGFR4*, rs374967242 in *FNDC1*, rs61754773 in *MMP8*, and rs188424977 in *FOXN1*) showed significant associations (OR, 3.89–37.3; p = 0.0058 to 0.0261) in combined analysis of the WES and replication samples (Table 2).

## Discussion

Recent GWAS identified some clear KD-associated loci and successfully identified part of the genetic background of KD; however, knowledge of these genetic factors has not made a significant contribution to understanding KD pathogenesis [12]. In this study, we performed WES with the aim of identifying rare protein-coding variants that contribute to KD susceptibility. We identified five rare coding variants in five genes (*FCRLA*, *PTGER4*, *IL17F*, *CARD11*, and *SIGLEC10*) associated with KD and another five coding variants in five genes (*FGFR4*, *IL31RA*, *FNDC1*, *MMP8*, and *FOXN1*) associated with CAA.

Among the five KD-associated genes, *FCRLA* has previously been reported to be highly significantly associated with low levels of affinity immunoglobulin gamma Fc region receptor II-a/b (products of *FCGR2A* and *FCGR2B*) in human blood (rs6668534: 0.434-unit decrease, p = 6 × 10^-17^) [18], as well as being associated with monocyte and lymphocyte counts [19]. *FCGR2A* is an established KD susceptibility gene [7,10]. These observations suggest that the effect of *FCGR2A* gene variants on KD susceptibility may be regulated by *FCRLA*. *PTGER4* is one of four identified receptors for prostaglandin E2 (PGE2). Previous studies investigated the function of PGE2 in relation to KD [20-23]. PGE2 levels are markedly elevated during the acute stage of KD and then decrease during the recovery stage [20]. PGE2 level was also correlated with prevention of IVIG resistance and CAA formation through CD40L [23]. Thus, *PTGER4*, a PGE2 receptor, may function in KD pathogenesis. The role of the *CARD11* gene in KD remains unknown; however, mutations of *CARD11* can cause a condition referred to as ‘B-cell expansion with NFκB and T cell anergy’, and immunodeficiency 11, due to defective B-cell receptor signaling [24,25]. *CARD11* is also associated with various immune-related diseases and traits, including multiple sclerosis, eczema, and platelet count [19,26,27]. Furthermore, defective B-cell development and B-cell functions are well known to be involved in KD pathogenesis [28,29]. Therefore, *CARD11* may play a role in susceptibility to KD. The roles of *IL17F* and *SIGLEC10* in KD susceptibility are unknown.

To date, several GWAS have been performed to identify susceptibility loci for coronary artery formation in KD, using a very limited number of patients with KD and CAA [30-34]. No overlapping genetic loci were detected from these GWAS. In this study, we identified five rare coding variants in five genes associated with CAAs in KD. None of these five genes, except for *MMP8*, have been reported to be associated with susceptibility to KD or CAAs in KD. *MMP8* encodes a member of the matrix metalloproteinase (MMP) family of proteins, which may be involved in maintaining the structure and function of coronary vascular walls [35]. An increasing number of studies have indicated elevated expression, activity, or protein levels of MMPs in KD [36,37]. *MMP8* expression levels are significantly upregulated in KD before IVIG treatment and decrease after undergoing IVIG treatment [37]. *MMP8* is also highly abundant in IVIG-resistant KD, suggesting that this is a potential biomarker for identifying those KD patients at highest risk for CAAs, and who may benefit from additional anti-inflammatory therapy [38,39].

To our knowledge, this is the first WES study of KD, and we identified several rare coding risk variants potentially associated with KD and CAA through WES and association analysis; however, we failed to confirm the significance of the selected SNPs in the replication study, mainly due to their extremely low frequency. Another reason for lack of significance in a replication study can be partly due to sample selection bias. To enrich for possible causative variants in the WES stage, we selected more severe KD cases than in the replication stage, such as patients with medium-sized or giant aneurysms (6.3% in WES samples vs. 1.7% in replication samples), IVIG resistance (50.3% vs. 22.5%), recurrence (14.5% vs. 5.5%), and family history (6.3% vs. 1.9%). Therefore, more variants that result in these severe phenotypes may have been discovered in the WES stage; however, in combined analysis of WES and replication data, statistically significant associations were detected. WES is a powerful technique for identification of protein-coding variants, which are more straightforward to annotate for biological functions and help to pinpoint causative genes; however, functional variants are more likely to be rare. Therefore, large sample sizes are usually required to detect loci with exome-wide significance, particularly for highly polygenic traits; however, we were only able to sequence a small number of samples, particularly in the case of children with CAA. Both common and rare variant association approaches are drastically underpowered to detect associations in small samples. Thus, our conclusions are tentative and preliminary, and further studies with large sample size are necessary to confirm our findings, as well as the functional role of the newly identified genes and variants in KD pathogenesis.

In summary, we sequenced the whole exomes of patients with KD and identified five candidate genes (*FCRLA*, *PTGER4*, *IL17F*, *CARD11*, and *SIGLEC10*) that may influence KD susceptibility, and another five candidate genes (*FGFR4*, *IL31RA*, *FNDC1*, *MMP8*, and *FOXN1*) with potential influence on the development of coronary aneurysms in KD. These results provide insights into new candidate genes and genetic variants potentially involved in the development of KD and CAA. Further association studies with expanded KD sample numbers from the Korean population or from cohorts with different ethnicities are required to confirm these results.

## Figures and Tables

**Table 1.** SNPs associated with KD

CHR	SNP	Gene	AA change	Allele^a^		WES (KD = 159 vs. Control = 902)				Replication (KD = 586 vs. Control = 732)				Combined (KD = 745 vs. Control = 1,634)			
						Genotype 11/12/22 (RAF)		OR	p	Genotype (RAF)		OR	p	Genotype (RAF)		OR	p
				1	2	KD	Control			KD	Control			KD	Control		
1	rs2275603	*FCRLA*	p.Ser209Gly	G	A	58/67/34 (0.575)	202/432/268 (0.463)	1.56	0.00023^b^	151/281/151 (0.500)	166/382/181 (0.490)	1.04	0.600	209/348/185 (0.516)	368/814/449 (0.475)	1.18	0.0088^b^
5	rs755244149	*PTGER4*	p.Arg358His	A	G	0/3/156 (0.009)	0/2/900 (0.001)	8.58	0.026^b,c^	0/3/580 (0.003)	0/1/728 (0.001)	3.76	0.329^c^	0/6/736 (0.004)	0/3/1628 (0.001)	4.41	0.031^b,c^
6	rs117796773	*IL17F*	Splice donor: c.254+1G>T	A	C	0/13/146 (0.041)	0/20/882 (0.011)	3.80	7.52E-05^b^	0/22/561 (0.019)	0/19/710 (0.013)	1.46	0.231	0/35/707 (0.024)	0/39/1592 (0.012)	2.00	0.0027^b^
7	rs41493047	*CARD11*	p.Ala687Val	A	G	0/9/150 (0.028)	0/13/889 (0.007)	4.01	0.00062^b^	0/12/571 (0.010)	0/11/718 (0.008)	1.37	0.453	0/21/721 (0.014)	0/24/1607 (0.007)	1.94	0.025^b^
19	rs201376644	*SIGLEC10*	p.Asn292Asp	C	T	0/18/141 (0.057)	0/22/880 (0.012)	4.86	7.91E-08^b^	0/4/579 (0.003)	0/1/728 (0.001)	5.02	0.109	0/22/720 (0.015)	0/23/1608 (0.007)	2.12	0.010^b^

Chi-square or Fisher’s exact tests were used, as appropriate. SNP, single nucleotide polymorphism; KD, Kawasaki disease; CHR, chromosome; AA, amino acid; OR, odds ratio; RAF, risk allele frequency.

a Allele 1 refers to a risk allele.

b Significant p-values.

c p-values of the exact test.

**Table 2.** SNPs associated with CAA (≥5 mm)

CHR	SNP	Gene	AA change	Allele^a^		WES (CAA = 26 vs. Non-CAA = 124)				Replication (CAA = 19 vs. Non-CAA = 420)				Combined (CAA = 45 vs. Non-CAA = 544)			
						Genotype 11/12/22 (RAF)		OR	P	Genotype (RAF)		OR	P	Genotype (RAF)		OR	P
				1	2	CAA^b^	Non-CAA^c^			CAA^b^	Non-CAA^c^			CAA^b^	Non-CAA^c^		
5	rs148721785	*IL31RA*	p.Val672Leu	C	G	0/6/20 (0.115)	0/5/119 (0.020)	6.34	0.0048^d^	0/1/18 (0.026)	0/18/400 (0.022)	1.23	0.574	0/7/38 (0.078)	0/23/519 (0.021)	3.89	0.0058^d^
5	rs201812753	*FGFR4*	p.Pro374Leu	T	C	0/4/22 (0.077)	0/1/123 (0.004)	20.6	0.0036^d^	0/0/19 (0.000)	0/8/410 (0.010)	NA	1	0/4/41 (0.044)	0/9/533 (0.008)	5.56	0.0135^d^
6	rs374967242	*FNDC1*	p.Trp59Arg	C	T	0/3/23 (0.058)	0/0/124 (0.000)	NA	0.0050^d^	0/0/19 (0.000)	0/1/417 (0.001)	NA	1	0/3/42 (0.033)	0/1/541 (0.001)	37.3	0.0017^d^
11	rs61754773	*MMP8*	p.Asp300Asn	T	C	0/3/23 (0.058)	0/1/123 (0.004)	15.1	0.0174^d^	0/1/18 (0.026)	0/10/408 (0.012)	2.23	0.389	0/4/41 (0.044)	0/11/531 (0.010)	4.54	0.0229^d^
17	rs188424977	*FOXN1*	p.Gly238Asp	A	G	0/3/23 (0.058)	0/0/124 (0.000)	NA	0.0050^d^	0/0/19 (0.000)	0/6/412 (0.007)	NA	1	0/3/42 (0.033)	0/6/536 (0.006)	6.20	0.0261^d^

Fisher’s exacts test was used. SNP, single nucleotide polymorphism; CAA, coronary artery aneurysms; CAA, coronary artery aneurysms; CHR, chromosome; AA, amino acid; WES, whole exome sequencing; RAF, risk allele frequency; OR, odds ratio; NA, not available.

a Allele 1 refers to a risk allele.

b Kawasaki disease (KD) patients with CAAs (internal diameter ≥ 5 mm)

c KD patients without CAAs.

d Significant p-values.
